# Supplementary figures and images for: Comparative transcriptome profiles of Schistosoma japonicum larval stages: Implications for parasite biology and host invasion
Source: PLoS Negl Trop Dis. 2022 Jan 13;16(1):e0009889. doi: 10.1371/journal.pntd.0009889 (PMC8791509; doi:10.1371/journal.pntd.0009889)

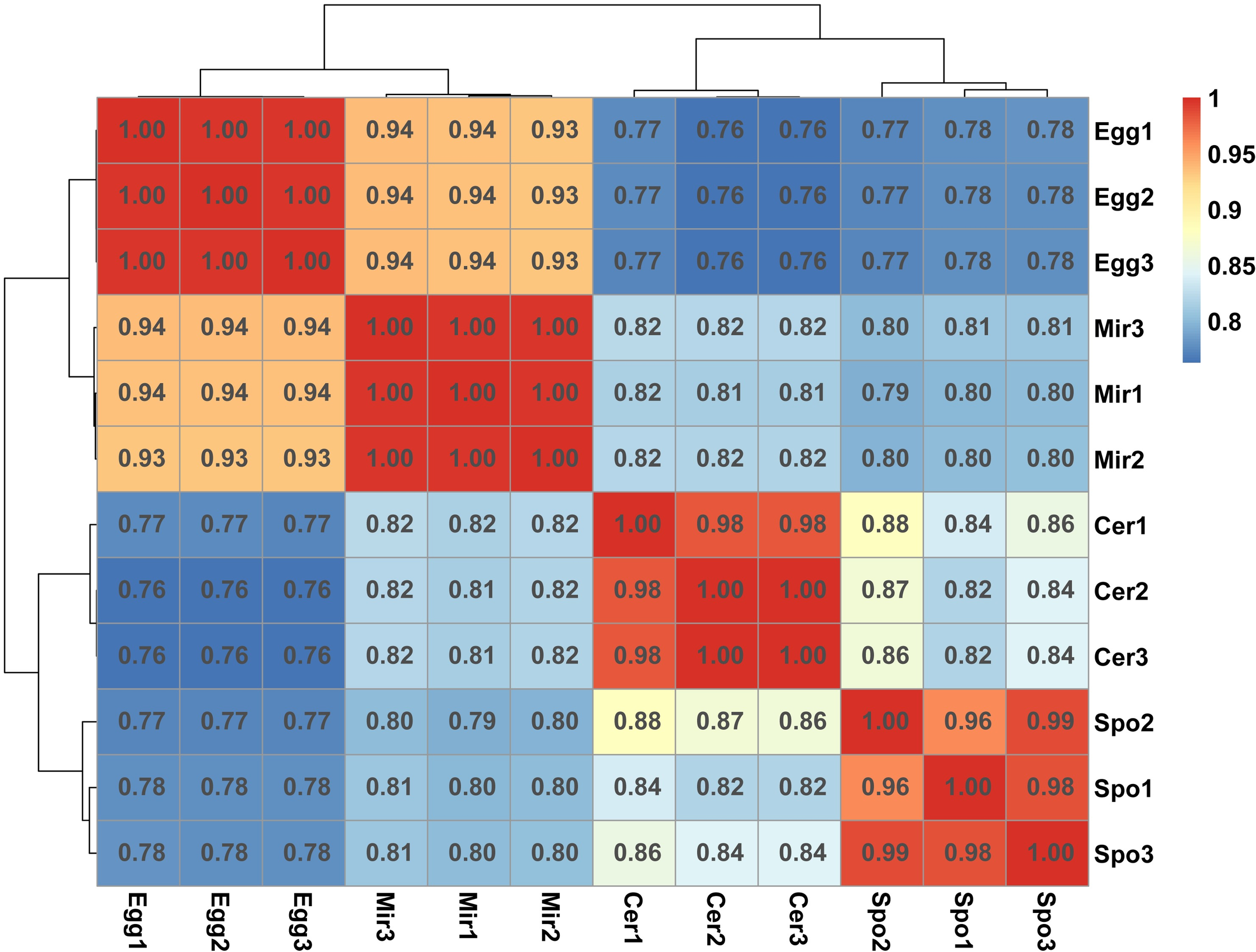

Supplement: S1 Fig — The scores calculated by the R function () indicated the correlation levels between two samples. (TIF) [file pntd.0009889.s001.tif]

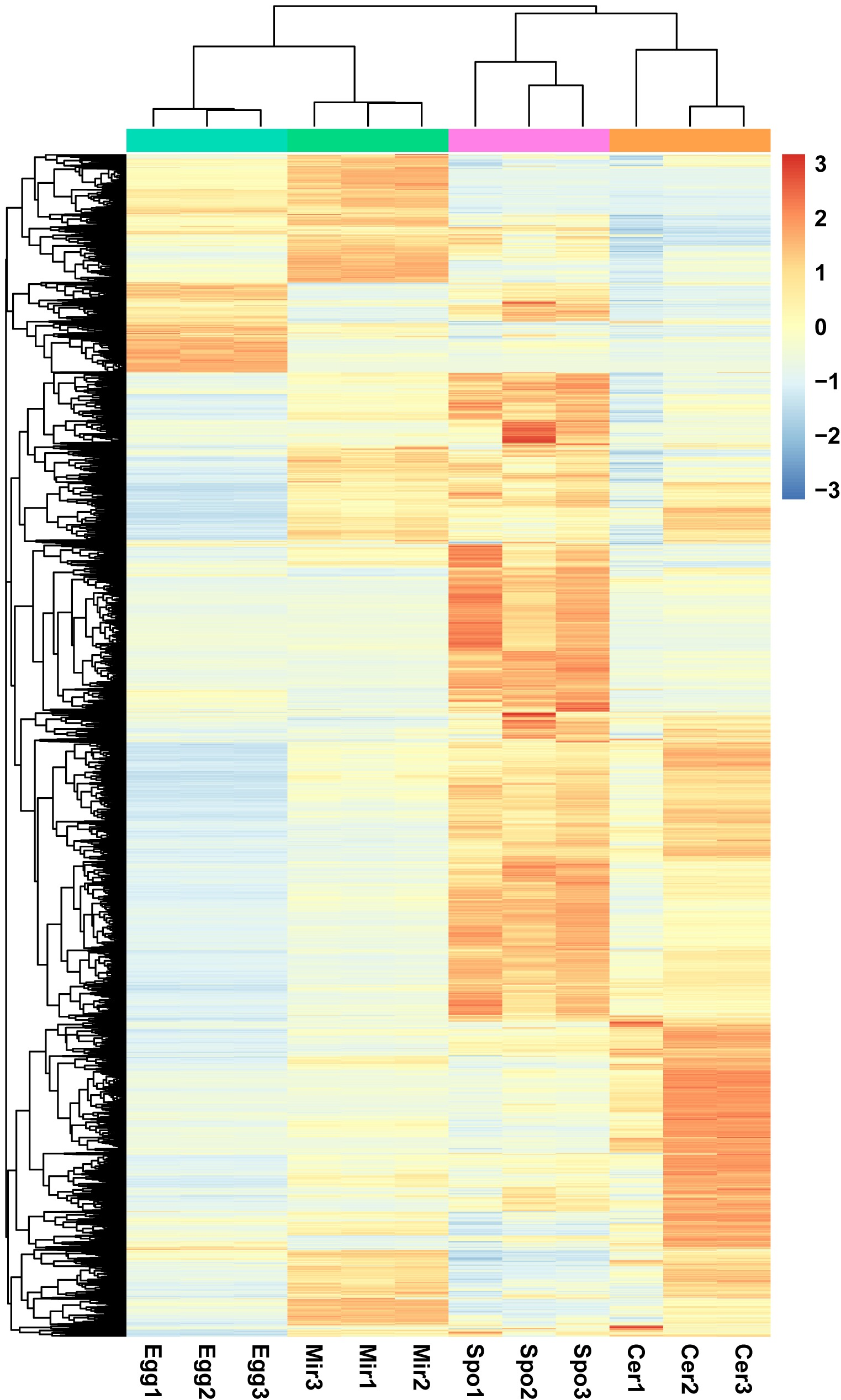

Supplement: S2 Fig — Egg, egg; Mir, miracidium; Spo, sporocyst; Cer, cercaria. (TIF) [file pntd.0009889.s002.tif]

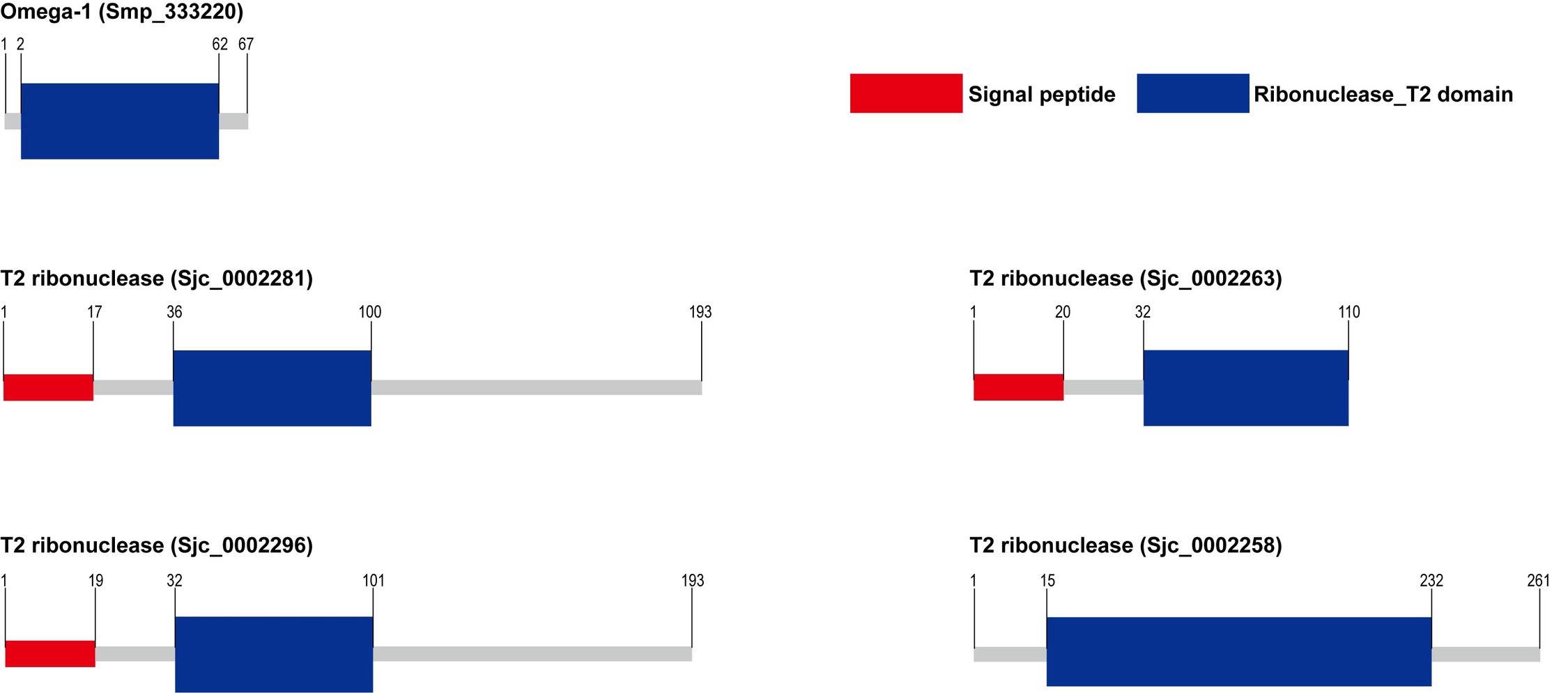

Supplement: S3 Fig — The signal peptide and ribonuclease_T2 domain are depicted in red and blue, respectively. (TIF) [file pntd.0009889.s003.tif]

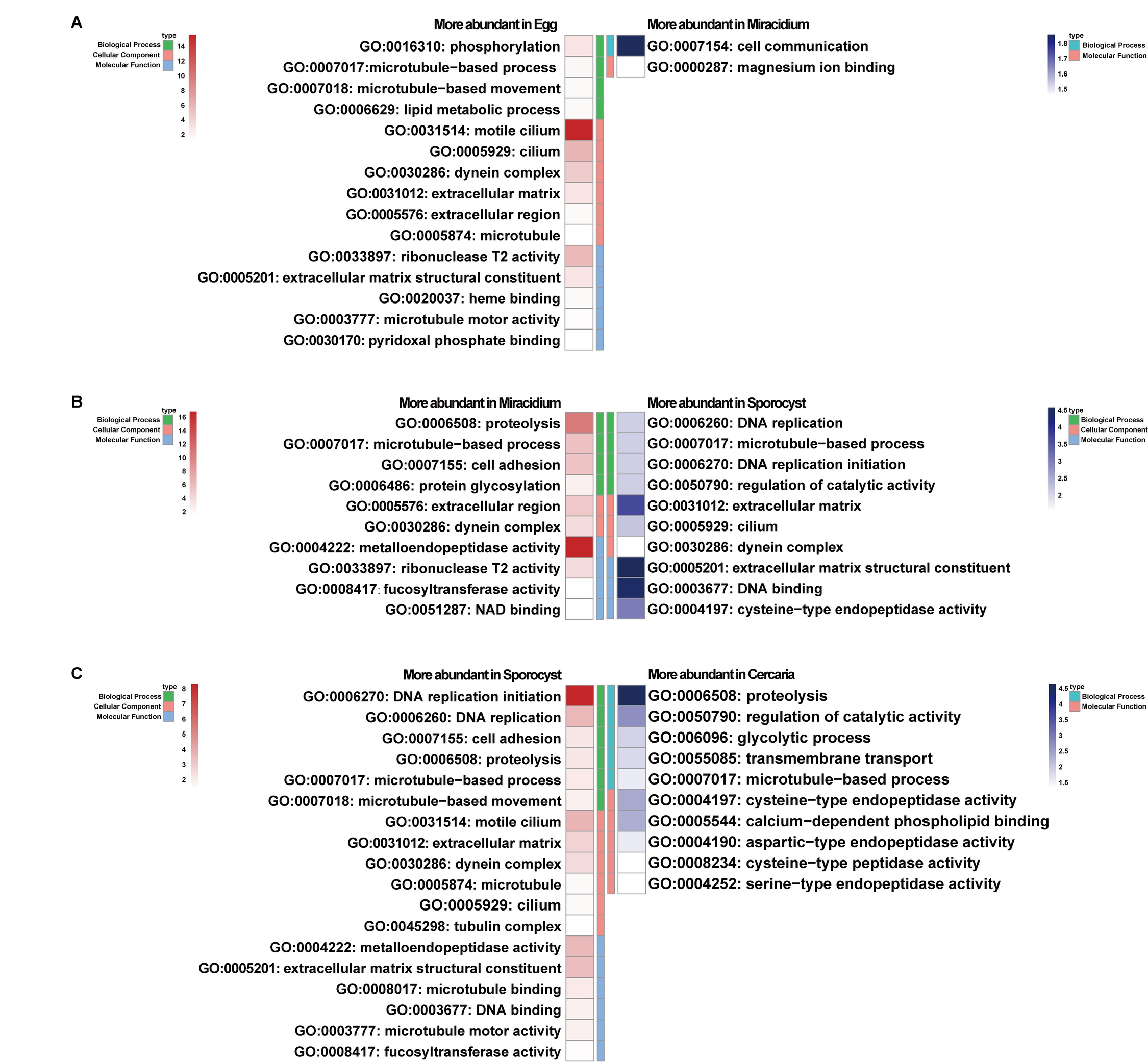

Supplement: S4 Fig — GO enrichment for differentially expressed genes (DEGs) in miracidium compared to egg (A), in sporocyst compared to miracidium (B), and in cercaria compared to sporocyst (C). (TIF) [file pntd.0009889.s004.tif]

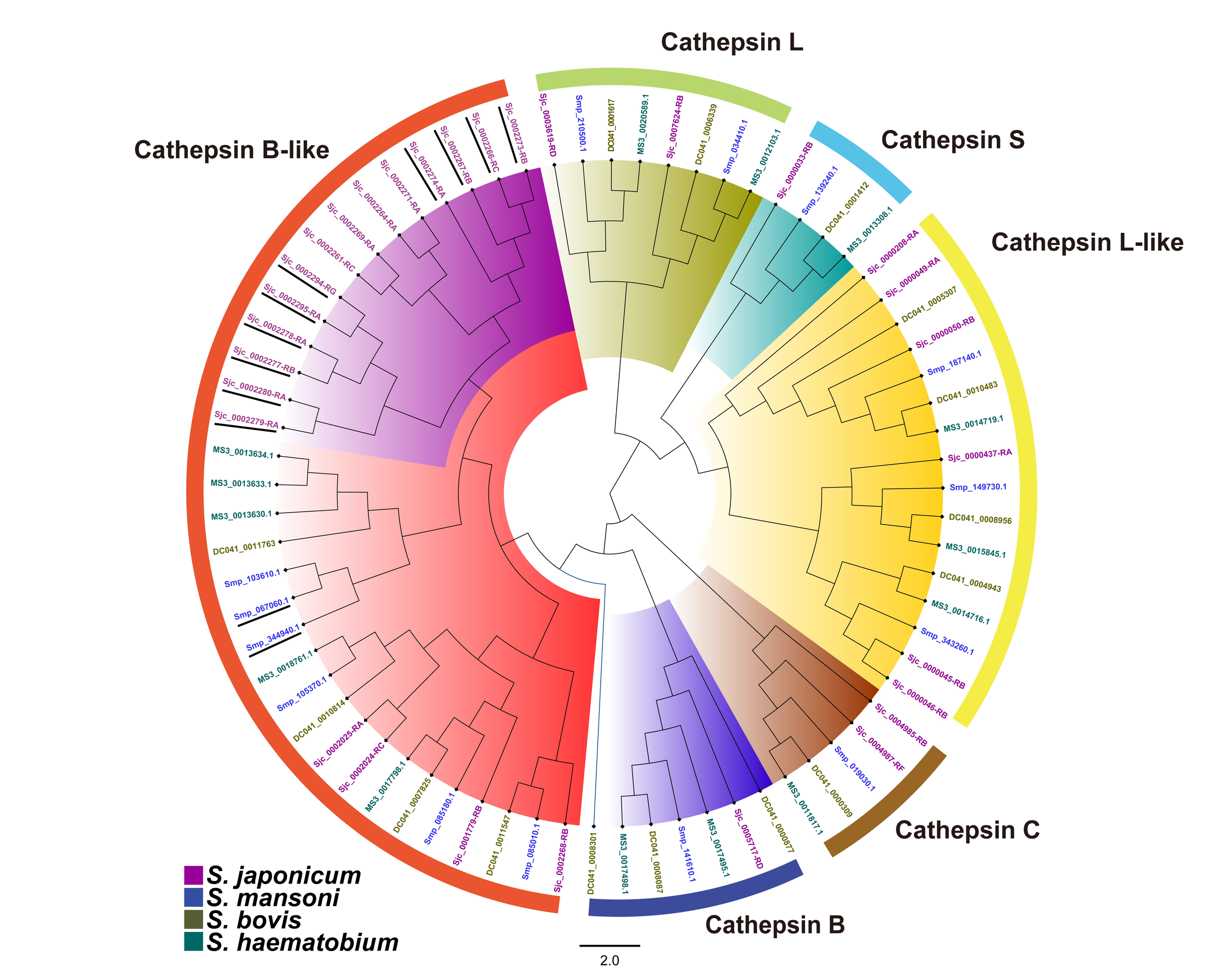

Supplement: S5 Fig — The protein and genome sequences of Schistosoma haematobium SchHae_2.0 [104] and Schistosoma bovis ASM395894v1 [105] were downloaded from the WormBase ParaSite (https://parasite.wormbase.org/index.html). An un-rooted phylogenic tree was constructed in MEGA 7 on the basis of multiple alignment of full-sequences from S. japonicum, S. mansoni, S. haematobium and S. bovis. Tandem duplicated cathepsin B-like cysteine proteases of S. japonicum and S. mansoni were indicated by bold black lines. (TIF) [file pntd.0009889.s005.tif]

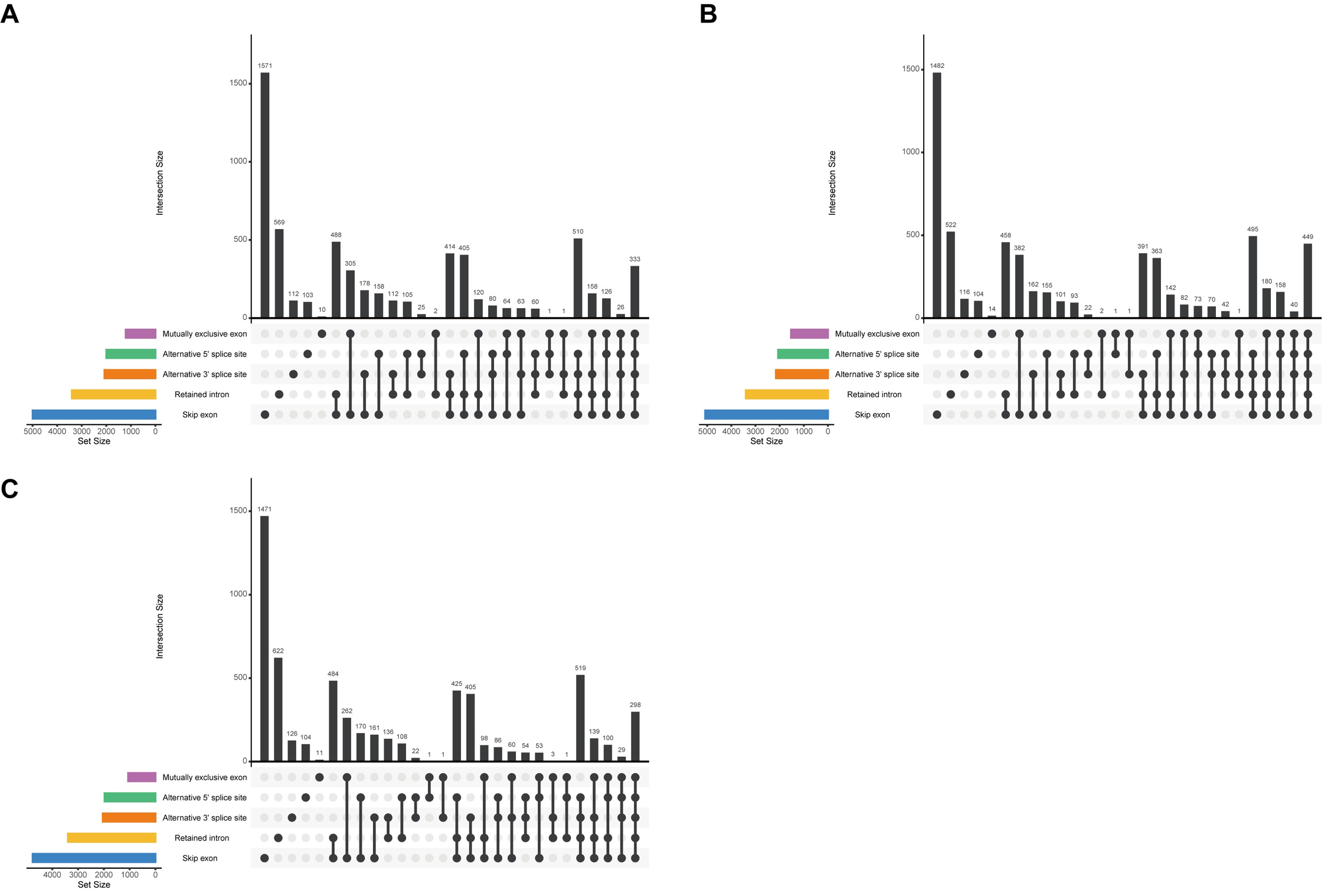

Supplement: S6 Fig — Interactions between the five types of detected AS genes in the (A) egg stage, (B) miracidium stage, and (C) sporocyst stage were visualized using an UpSet plot. (TIF) [file pntd.0009889.s006.tif]

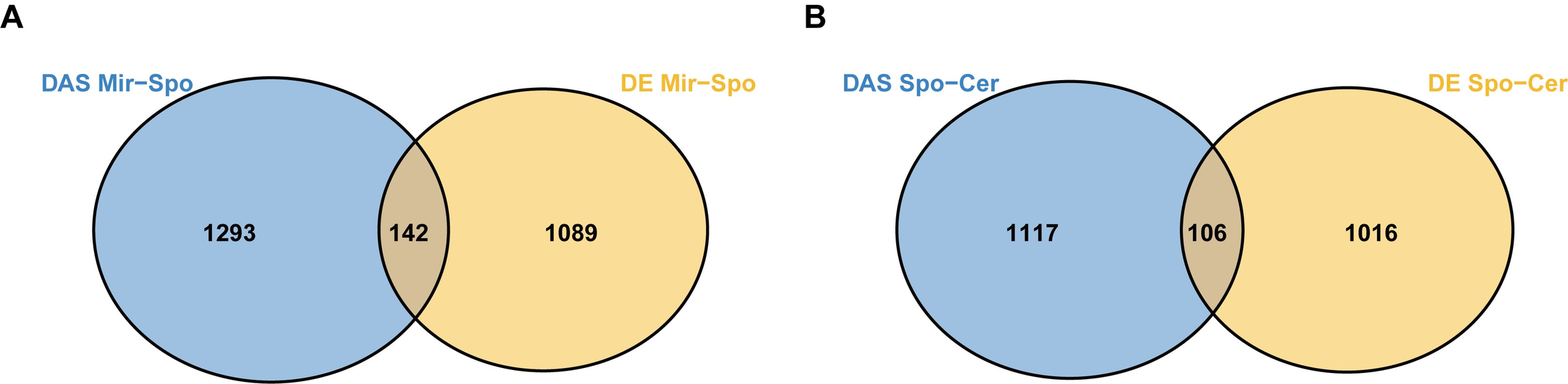

Supplement: S7 Fig — Venn diagram of the overlap of the DE and DAS genes between the (A) miracidium and sporocyst stages, and (B) sporocyst and cercaria stages. (TIF) [file pntd.0009889.s007.tif]
