# Supplementary material for: Comparative transcriptome profiles of Schistosoma japonicum larval stages: Implications for parasite biology and host invasion
Source: PLoS Negl Trop Dis. 2022 Jan 13;16(1):e0009889. doi: 10.1371/journal.pntd.0009889 (PMC8791509; doi:10.1371/journal.pntd.0009889)
Supplement: Attachment — Submitted filename: Response to Reviewers.docx [file pntd.0009889.s018.docx]

Reviewer Comments to Author:
Methods

Reviewer #1:

*1. Could the authors include a comment on how the cooling of cercariae prior to centrifugation may impact the transcriptome of this life cycle stage.*

Response: We believe cooling could induce the transient transcriptional changes in cercariae for some extent. Unfortunately, this is the standard method to collect these tiny and highly motive cercariae.

*2. Egg isolation and hatching methodology – lines 134-37.*

Response: Details for egg isolation and hatching methodology has been added in the revised manuscript (lines 149-172).

*3. How were the repeat experiments performed? e.g. were there independent infections/hatchings?*

Response: In this study, we collected samples for each stage from three biological replicates. And they were all from independent infections/hatchings. E.g., eggs isolated from one rabbit were used as one replicate. We have modified the “Parasite material’ section by adding details of these samples (revised manuscript, lines 149-184).

*4. The amounts/volumes of parasite material used for RNA extraction.*

Response: For the RNA extraction from each sample, we used 100~150 mg eggs, 15, 000~20, 000 miracidia, 1~2 mg daughter sporocysts, and 15, 000~20, 000 cercariae. We have added this information into the revised manuscript (lines 187-189).

*5.  Provide details of the input parameters used for each bioinformatic analysis. E.g. the read trimming criteria used with the fastp tool.*

Response: Both the input parameters and version number of the bioinformatic software were provided in our revised manuscript.

*6. Provide references for all bioinformatic tools used.*

Response: References for all bioinformatic used were provided.

*7. Authors write that the genome edition used here is currently under review elsewhere, once published they should update this manuscript with the appropriate reference.*

Response: Thanks for your understanding. All the analyses in this manuscript were based on *Sj*V3 genome. We have corrected the confusing statements.

*8. I couldn’t find a clear description of the methods/tools/parameters for clustering genes by expression -for data shown in Figure 2D.*

Response: The genes were clustered using R package Mfuzz v 2.46.0. In the revised manuscript, we provided the methods/tools/parameters for gene clustering (lines 220-225).

*9. For the Peptidase C1 identification and phylogenetic analysis section: authors only mention the 2-species analysis, not the 4-species included in the supplementary figures.*

Response: Thanks for your careful check. We have added the four species analyses in the figure legend of S5 figure (revised manuscript, lines 1072-1079).

Reviewer #2:

*Appropriate methods used, all very clearly described.*

Response: Thank you!

Reviewer #3:

*1. Yes, the life stages assessed are appropriate. Harvesting miracidia from “artificial pond water” (whatever that is?) sounds fairly reasonable, dissecting sporocysts from snail sounds a bit labour intensive but appropriate, and cercariae harvesting followed by rapid cooling sounds like a good plan. In RNA-Seq it is always very tricky to harvest the right tissue in the right stage, in as “normal” setting as possible. For example, larvae exposed to tap water might have very different expression profile to larvae exposed to actual pond water.*

Response: Thanks for your positive evaluations. The protocol of artificial pond water (Milli-Q water plus 0.46 µM FeCl_3_ ⋅6 H_2_O, 220 µM CaCl_2_ ⋅2 H_2_O, 100 µM MgSO_4_ ⋅7 H_2_O, phosphate buffer [313 µM KH_2_PO4, 14 µM (NH_4_)_2_SO_4_] pH 7.2) is from this reference [1]. We used artificial pond water in the egg hatching and cercariae shedding processes. The reference and the composition of the artificial pond water was added in the revised manuscript (lines 164-167).

*2. The description is quite clear, with some exceptions like “artificial pond water”. The samples are not quantified, so we don’t know if the RNA contains 1,10 or 100 individuals.*

Response: We have provided the formula of the “artificial pond water”. For the RNA extraction from each sample, we used 100~150 mg eggs, 15, 000~20, 000 miracidia, 1~2 mg daughter sporocysts, and 15, 000~20, 000 cercariae. We have added this information into the revised manuscript (lines 187-189).

Results

Reviewer #1:

*1. Gene expression information correlated well with the well-described biology of each life stage’ (Line 114). Here a brief summary of what aspects of the biology are relevant/interesting here would help the reader. Were there changes the authors expected/predicted to see? The same applies to Line 243.*

Response: A brief summary of the biological characteristics of each stage were added. Please check our revised manuscript for the details (lines 120-124 and 312-316). We expected and have seen the cell division at the sporocyst stage.

*2. GO enrichment analysis was used to interrogate the differences between samples. I think the analysis would be improved by also looking for Pfam enrichment (or similar) as GO coverage is variable in parasite genomes.*

Response: Thanks for your suggestion. GO enrichment analysis have been widely used in transcriptome studies of *Schistosoma* [2–4], but Pfam enrichment analysis is rarely used. Moreover, based on the ever-enriching databases and our *Sj*V3 genome, the GO enrichment results in this study seem quite well.

*3. Reported putative stage-specific leishmanolysin expression (line 347). Are there sequence/functional/localisation differences between these groups of genes that might offer a hypothesis for this?*

Response: There are two groups of leishmanolysins in our main text, one is specifically expressed in sporocysts and the other one is downregulated during the development from miracidia to sporocysts. These two groups of leishmanolysins were closely related in sequences, but we don’t know their function or localization.

*4. Lines 363-365 – vague*

Response: To make it clear, we have rephrased the sentence as ‘Many members of these important protease family have been studied, such as cathepsin B and cathepsin L from cysteine protease family [5], cathepsin D from aspartic protease family [6] and trypsin and elastase from serine protease family [7]. These proteases participate in numerous biological processes, such as the parasite's invasion, survival and longevity in the definitive host.’ (revised manuscript, lines 469-474).

*5. Gene clustering based on transcript abundance (lines 368-392 and figure 2D) – the clusters for groups 3/4 seem quite similar to me in the figure. Can the authors discuss why they are separate clusters?*

Response: Thanks for your comment. We applied the fuzzy c-means algorithm to soft cluster gene expression profiles in these four life stages. After different parameters were tested, the cluster number was manually set at c=8. Although groups 3 and 4 seem quite similar, but they were divided into different groups based on the algorithm. The difference between the two groups is the gene expression level in the cercariae stage. As shown in Fig 4, genes in group 3 showed higher expression levels than those in group 4.

*6. Lines 385-386 – vague.*

Response: Sorry for the confusion. This sentence has been reworded as ‘This may reflect that parasites at these two free-swimming stages are preparing proteins used for the host invasion.’ (lines 503-504).

*7. It would be good to include the HMMER output statistics for the peptidase C1 (including p-values) and sequence alignments – likely as a supplementary figure. That would help the authors address the following: How similar are the genomic/predicted protein sequences for the tandemly duplicated C1 peptidases? In relation to this, can the authors comment on the mapping statistics for reads over the tandemly duplicated genes? Are the reads uniquely mapped to a single copy?*

Response: Thanks for your reminding. We have provided the HMMER output statistics and multiple sequence alignments for the peptidase C1 (papain) in Dataset S1. As shown in the BLAST result (Dataset S1), the C1 peptidases (papains) exhibited an overall similarity between 78.73% and 99.46%. For the mapping statistics, usually it is designed for the reference transcriptomes instead of specific genes. We have similar concerns about the quantification of the tandem duplicated C1 peptidases (papains) and chose RSEM, because it can handle reads that maps ambiguously (multiple-mapped reads) to achieve the best estimation accuracies with a statistical model.

*8. The alternative splicing (AS) analysis raw data tables using to make figure 4A should be included in the supplementary, including p-values and gene IDs of alternatively spliced genes. This analysis is simply stated with little discussion. One gene is highlighted without justification in Figure 4F. Authors briefly compare the types of AS in larval stages and adults (line 502), more information to summarize the findings in adult stages would be useful to the reader here.*

Response: We have provided the raw data tables of AS in each life stage as well as the differential AS events in the three developmental transitions including *p*-values and gene IDs in these tables (Dataset 2). The highlighted gene in Figure 4F was just taken as an example to show the differential AS events occurred in the adjacent life stages so that we did not speculate its potential function. We have added the summarized findings in schistosomula and adult stages as you suggested (revised manuscript, lines 676-680).

*9. Line 73 – The statement that this work provides functional information for S. japonicum is slightly overstating results. I would suggest rewording this*.

Response: Thanks for your advices. This sentence has been modified as ‘Our data provide valuable information on the expression of *S. japonicum* genes across these life stages and will support basic and applied research for the community.’ (revised manuscript, lines 74-76).

*10. Lines 252-259 –A direct link between Omega-1 function and the differentially abundance putative* *T2 ribonucleases is unclear. As such I think authors need to be more cautious in the wording here. If there are additional references/evidence supporting these speculations, they should be included. E.g. Is there evidence for function validation/co-localisation/co-expression of these putative ribonucleases with Omega-1?*

Response: In this work, we found four *S. japonicum* T2 ribonucleases were specifically expressed in eggs. Omega-1, as a member of T2 ribonuclease family, was well-known in *S. mansoni* for its function in regulating host immune response. Here, we compared the protein structure of Omega-1 and four *S. japonicum* T2 ribonucleases and found that they all contained the ribonuclease_T2 domain (S3 Fig). Additionally, we cited a new reference (reference 31) that reported all copies of *S. mansoni* omega-1 were specifically expressed in eggs which further supported our speculation. We have added more information and made corrections to this sentence in the revised manuscript (lines 325-341).

*11. Line 289 - reference 40 appears to be a transcript focused paper and does not verify correlation of protein and transcript abundances here, consider rewording.*

Response: Thanks for your careful check. Reference 40 provides the information that genes encoding penetration enzymes were transcribed in the germ ball (see the ‘Summary’ section, lines 9-11). To avoid confusion, we mortified the sentence as ‘It's worth noting that the cercaria head is mostly transcriptionally and translationally quiescent [8]. The elastase and leishmanolysins transcribed in the daughter sporocysts may be prepared for the cercariae penetration.’ (revised manuscript, lines 374-377).

*12. Line 317 – it is not clear to be that the statement ‘CaBP may be essential for the infectious cercaria’ is well justified.*

Response: CaBP (*Sjc*_0003236) is the most enriched gene at the cercaria stage, this information has been added into the revised manuscript (lines 397-399). Combining its high expression in cercariae and the studies of CaBP in trematodes [9,10], we speculate that CaBP may play important roles in the cercariae stage. This sentence has been rephrased as ‘These findings suggest that the function of calcium may be conserved among trematode species by regulating the larval physiology, while CaBP may be important for the infectious cercariae.’ (lines 407-409).

*13. Line 329 - The reason for the use of a foldchange cut off > 5 should be stated.*

Response: We used a foldchange cut off >5 to obtain more representative GO terms. This information has been added in the revised manuscript (lines 427-428).

*14. ‘These data indicate that leishmanolysin and neprilysin maybe involved in the penetration of the snail host by the miracidium.’ – not well justified, consider rewording.*

Response: Thanks for your comment. It was revealed that one leishmanolysin from *S. mansoni* ES products could interfere with the migration of *Biomphalaria glabrata* hemocytes [11] (referenced in the manuscript). Besides, neplilysin was shown to involve in the production of immunoactive peptides, which could inactive the immunocytes from the snail host [12]. This sentence has been reworded as ‘These data indicate that leishmanolysin and neprilysin may help the parasite escaping the immune attacks of the *Oncomelania hupensis* hemocytes after miracidia penetration.’ (revised manuscript, lines 456-458).

*15. The use of subheadings might help the flow of the ‘**Stage-specific genes (SSG) and stage-enriched genes (SEG)’ section.*

Response: Four subheadings of this section were added in the revised manuscript, they are ‘SSG and SEG in eggs’, ‘SSG and SEG in miracidia’, ‘SSG and SEG in sporocysts’ and ‘SSG and SEG in cercariae’.

*16. Where specific genes are mentioned the gene IDs should be put in the text too. E.g. Lines 299-300 and 269 etc.*

Response: Thanks for your reminding. Gene IDs of all mentioned specific genes were added.

*17. Line 254 – unexplained acronym ‘SEA’.*

Response: ‘SEA’ refers to ‘soluble egg antigen’. We have added this annotation in the revised manuscript (line 325).

*18. Line 307 – This GO term appears to be wrong. I think it should be 5509.*

Response: Corrected.

*19. Authors use ‘associated with the transition from’ a few times (e.g. line 325,342) I think this isn’t strictly accurate. Consider rewording.*

Response: The word ‘transition’ has been replaced with ‘development’. All the inaccurate wording have been corrected (revised manuscript, lines 422, 430, 446, and 464).

*20. The authors could take greater care with some of the wording in their Results/Discussion regarding use of ‘expression’. The work describes the larval stages transcriptomes and transcript abundances do not always correspond to protein abundance. It is suggested that the authors use more conservative terms like ‘transcript abundance’ rather than ‘gene expression’.*

Response: Thanks for your comment. ‘Gene expression’ is broadly used in RNA-seq papers to describe the ‘transcript abundance’ [2,13,14].

*21. I would suggest moving the supplementary figures 3A, C, E to the main figures as these plots nicely describe the data. Similarly, I would recommend moving 4B to the supplementary figures. (Not required, merely suggestion).*

Response: Thanks for your suggestions. Supplementary figures 3A, C, and E were moved into the main figures and become the new Figure 3. Figure 4B (the new Figure 7B) was still in the main figures, because it can nicely show the proportion of different AS type in each life stage.

*22. Figure 1A – ‘PCA results’ is not descriptive enough here.*

Response: More descriptions have been added to the PCA results (revised manuscript, lines 283-286).

*23. Figure 1C – clarification of ‘differentially expressed’ and ‘commonly’ expressed as this was not immediately clear.*

Response: Thanks for your comment. Legend of Fig 1C has been corrected into ‘Venn diagram showing differentially transcribed genes among the four life stages. Circles of different colors represent a set of genes transcribed in one stage, where the values represent the number of uniquely transcribed genes in one stage, or common transcribed genes between two, three or four life stages.’ (revised manuscript, lines 305-309).

*24. Figure 2A – On figure, ‘Specifical’ change to specific?*

Response: Corrected.

*25. Figure 2B – I found this figure difficult to interpret with the GO accession numbers on the plot, I would recommend putting these with the text to the left. What is mean by the ‘Gene Ratio’ should be stated in the legend, additionally in the legend for Table S2.*

Response: Thanks for your suggestion. The GO accession numbers have been added to the Figure 2B with the text. Gene ratio is the percentage of total SEG or SSG in the given GO term. The definition of gene ratio has been added in the legend of Figure 2B and S3 Table.

*26. Figure 2D – ‘Membership’ metric was not well explained. State y-axis units.*

Response: Membership value was calculated using R package Mfuzz, which indicated the degree of membership of this gene for the corresponding cluster. The y-axes ware scaled independently to emphasize the differences between clusters. We have added this information in the legend of Figure 4 (revised manuscript, lines 506-509).

*27. Figure 3B – Missing units on x-axis bar.* *Clustering on right of figure squashed making it difficult to see clusters – this may benefit from labels indicated the discussed clusters. The coloring of motifs/genome features clashes a bit, can you make them more distinct?*

Response: Thanks for your careful check. The units on the x-axis were added (left: amino acids; right: nucleotides (bp)). The labels were also added to indicate the discussed clusters (see the new Figure 6B). We used TBtools software to visualize the analysis results with default parameters and color palette. This software doesn’t support the user-defined color settings; thus, we keep the coloring of motifs/genome features.

*28. Supplementary Figures 1 and 2 – label the legend in the figure to clearly indicate what score the color gradient is reflecting. More detail is needed as to how these scores are arrived at, either in the figure legend or in the Materials and Methods section.*

Response: The correlation coefficient was added in the revised S1 Figure. The scores indicated the correlation levels between two samples. This information and the method used for calculate the Person’s correlation coefficient were added in the legend of Figure S1 (revised manuscript, lines 1056-1058). We don’t think it’s necessary to add the gene expression values in the S2 Figure, because there are a total of 8,732 genes, even if they are added, they are not visible.

*29. Supplementary Figure 3
For A/C/E - some points are cut off from the plot, the y-axis should be adjusted. Include what the dotted lines represent in the legend. Additionally, the axis scale appears to be Log2 foldchange but the highlighted datapoints are based on fold change – this was a little confusing. Check point highlighting for p-values, some non-significant points appear to be coloured.*

Response: Thanks for your careful check. S3 Figure A/C/E were moved into the main figures and became the new Figure 3. It was worth noting that the p-value of some genes were nearly 0, so the -log10(Adjust P-value) were nearly ∞. Therefore, we defined the Adjust P-value of these genes as 10^-500^ (-log10(Adjust *P*-value) =500), then these points can be included in the plot. The dot line on the y axis refers to *p*-value =0.05. The dot lines on the x axis refer to fold change = -2 or 2. The scale of the x axis was Log2FoldChange, and we have made conversions. All the non-significant points were colored in grey. These details have been added in the legend of the new Figure 3.

*B/D/F – the legends are quite small making them difficult to read. GO term numbers would be useful to add to these also.*

Response: The S3 Figure B/D/F were changed to the new S4 figure A/B/C. The texts of the new S4 Figure were adjusted to make them easier for reading. The GO term numbers were added. See our the new S4 Figure for more details.

*3A –* *Can the authors comment on the skew to down negative fold changes show in the plot?*

Response: Thanks for your comment. There are no much differences in the numbers of the upregulated or downregulated genes during the development from miracidium to sporocyst, or sporocyst to cercaria. During the development from egg to miracidium, much more genes were downregulated. Many of these genes may be involved in the miracidium development, and some are egg specific, like the egg protein CP422 (*Sjc*_0009568).

*3B – there is no discussion of the magnesium ion binding GO enrichment in Miracidium the main text. Not essential but consider discussing.*

Response: Add discussion. The GO term ‘magnesium ion binding’ enriched in miracidium was discussed in the revised manuscript (lines 430-435).

*30. Figure 4F – including the structure (exons, introns etc.) of the whole transcript or genomic sequence at the base of this plot would aid interpretation of the isoforms shown.*

Response: We have labeled the exons and introns in the new Figure 7F to aid the interpretation of the isoforms shown. It is worth noting that this plot didn’t show the whole gene structure, but only the part that underwent alternative splicing.

*31. Figure S4 – are all duplication events indicated on this tree? I think the S. mansoni ones from the main figure are missing. It would also be good to add in any from the two additional species.*

Response: Thanks for your careful check. Duplication events of *S. mansoni* were also indicated by bold black lines (see the new Figure S5). Considering the current genome assembly of *Schistosoma haematobium* and *Schistosoma bovis* haven’t reach the chromosomal level, we didn’t analyze the tandem duplication of the peptidase C1 genes in these two schistosomes.

*32. Finally, I would encourage the authors to consider including a graphical abstract/figure highlighting the main findings in a schematic of the life cycle stages. This would help communicate the key findings and summaries the work.*

Response: We would like to summarize the main findings in the final version.

Reviewer #2:

*Excellent and thorough presentation of results*

Response: Thank you!

Reviewer #3:

*1. Yes, it does. All results reported have clear methods sections which explain how they got there, and their choice of analysis methods are overall appropriate. The one bit that is missing is a method/cut-off for alternative splicing and alternative transcript reconstruction. I just cannot follow how they go from mapped reads to predicting alternatively spliced gene models, to determining if there are significantly different transcript expression patterns between life stages.*

Response: Thanks for your comment. We quantified alternative splicing events using rMAts-turbo with the default parameters, rMATS-turbo will take data about where reads align and where alternative exons are to determine how often an exon is included in a sample. It will quantify this using a metric called PSI (Percent Spliced In). rMATS-turbo will then compare PSI values between replicates and across experimental conditions to identify alternative splicing events whose PSI values are significantly different across conditions. For more details, please visit this website: https://github.com/Xinglab/rmats-turbo.

*2. The PCA plot looks good, and supports good data quality – although the biological replicates look very very similar to each other. The choice of figures, and the supplementary tables are excellent. It might have been nice to have a table of all read counts/FPKMs? But apart from that all you expect is there.*

Response: Thanks for your comment. A table of all TPMs was provided (revised manuscript, S2 Table).

Conclusions

Reviewer #1:

*1. I could not find a clear discussion of the limitations of the study – this would be useful to address comments relating to the methods section e.g. cooling of cercariae during harvesting. I would also be interested to read about any ideas the authors had for priorities and next steps from this work in the concluding remarks.*

Response: We are very grateful for your careful check of our manuscript. All of your questions were thoroughly answered above, including the method of cooling the cercariae. We are planning to study the functions of the proteases that involved in the penetration of the intermediate and definitive hosts. At this moment, we are focus on the leishmanolysin and its role in the *S. japonicum* cercariae invasion process.

Reviewer #2:

*Conclusions are appropriate and supported by data.*

Response: Thank you!

Reviewer #3:

*1. Some of the conclusions feel a bit speculative, eg. 261-275 the authors suggest that CFAP could either be “involved in the assembly of the cilium” or involved in chemical detection of the snail host, based on CFAP role in mice. Those are both fine hypotheses, which could be tested, but seems a bit speculative to present as a result for this present study.* *In contrast, the discussion 270-275 about 5-HT contains references to much more relevant and specific studies. The results 277-296 also contains relevant references, and suitable comparison to similar species and similar results, however, it only shows that what is seen in S. japonicum is exactly what is expected, and has been known for >10 years.*

Response: Thanks for your comment. Because these life stages are very tiny and the research methodologies are limited in these stages. We can only speculate their possible roles in these larval stages based on the studies of the homologs in other species. Of course, these speculations need to be further verified by experiments. We have added some new content in the ‘SSG and SEG in sporocysts’ section (revised manuscript, lines 377-383). Besides, we have added a new part named ‘Genes associated with germinal cell and asexual reproduction in the larval stages’ in the revised manuscript (lines 511-567). We found that stem cells are existed in every larval stage and there maybe stem cell conversion during the larval development.

*2. The very nice section about tandem genomic events in C1 (394 ff) is interesting, but the main take-home is the argument that several cathepsins have undergone genomic duplication. And since this paper is not publishing a new S. japonicum genome, or even making one publicly available, it is very hard to validate this section. S. japonicum genomes have also been published before, and it would be nice to be offered a view into if their findings can also be seen in the prjna520774 genome assembly. Maybe this whole section would fit better into the genomic paper, because the final take-home message relating to expression data in fig 3d is not novel, but merely a confirmation what is already known. Not to nit-pick, but the HMMer analysis of PF00112 has already been pre-calculated for you in Wormbase ParaSite if you go to the BioMart tool (which shows 10 C1-containing proteins, rather than the 29 or 30 reported by the authors – and the lower number is more in line with what is expected from S.mansoni. Of course we cannot know for sure which is right, and it is possible that the current S. japonicum genome assembly and annotation is lacking, and it would be worth trying to tease it out which is more right. But this paper does not convince me that they have done all the job needed to prove it either way. I do look forward to the genome publication coming out, to see if this question has been addressed more in-depth there – which would in my opinion be the more appropriate place for that whole section.*

Response: Thanks for your comment. First of all, the *Sj*V3 genome has been uploaded to the NCBI database (BioProject: PRJNA739049). Because the paper of the *Sj*V3 is still under review, so we are unable to release the data right now. We have provided the GFF file of peptidase C1 to show their locations in the *Sj*V3 genome (Dataset S1). According to your proposal, we have searched the *Sj*V2 genome (PRJNA520774) and found that there were actually 26 peptidase C1 genes in total. The HMMER output statistics and the annotations of the peptidase C1 genes in *Sj*V2 were provided in the Dataset S1. Secondly, we don’t know why only ten C1 peptidases were identified by the BioMart tool, but some C1 peptidases were definitely missed. EWB00_001523, a cathepsin B-like cysteine protease, was showed in our result but not identified by the BioMart tool. Our analysis pipeline has been described in many publications [15–17]. We identified the peptidase C1 genes by using the HMMER program with a *p*-value cut-off of 1e-5. Finally, we agreed that the title of this section may confuse the readers, so that we change it to ‘Highly expressed cathepsin B-like proteases in cercariae were produced by tandem duplication’. The goal of this work is to uncover the comparative transcriptome profiles of *S. japonicum* larval stages for better understanding of the parasite biology using RNA-seq. In this analysis, we observed genes belong to peptidase C1 family were highly expressed at the cercariae stage. Moreover, the cathepsin B protease, which has been reported to be involved in *S. japonicum* cercariae invasion in our previously studies [18,19] is one of them. Given that, we hypothesized peptidase C1 family play an important role in the cercariae invasion. So we performed genome-wide analysis of peptidase C1 gene family in *S. japonicum* and found *a* group of cathepsin B-like genes that had high expression at cercariae stage were produced by tandem duplications. A further study on the functions of members in peptidase C1 gene family should be performed but out of the scale of this work.

Editorial and Data Presentation Modifications

Reviewer #1:

*I have included comments relevant to this section in the ‘Results’ section as I felt they were clearer there.*

Response: Thanks for your comment, all your questions in the ‘Results’ section have been answered, please see our answer above.

Reviewer #2:

*N/A*

Reviewer #3:

*1. Minor: In the methods section, expand a little bit on how many individuals were combined into each sample? Also – how were the biological replicates made? Eg parasites extracted once, and split into three samples, or taken 3 times on different days?*

Response: Thanks for your careful check. In this study, we collected samples for each stage from three biological replicates. And they were all from independent infections/animals. E.g., eggs isolated from one rabbit were used as one replicate. We have modified the “Parasite material’ section by adding details of these samples (revised manuscript, lines 149-184).

*2. Minor: Throughout the text there are a few instances of abbreviations being introduced without being spelled out, eg line 254 “SEA”, which authors might want to clarify in this context means “soluble egg antigen”. “C1” that is referred to multiple times in the text might be known to more readers as “Cysteine peptidases” or more specifically cathepsin, which is of large importance due to their role in schistosoma vaccine research eg* *PMC3897446, and the relevance of those were not introduced in the introduction, although they feature in the discussion. Minimising abbreviations makes the text more readable.*

Response: SEA refers to ‘soluble egg antigen’. We have added this annotation in the revised manuscript (line 325). The peptidase C1 (papain) family is part of clan CA of cysteine peptidases. Most of the ‘peptidase C1’ were replaced with ‘papain’ to make the context more readable. We have cited PMC3897446 (reference 85) and emphasized that calpains can be good vaccine targets in the revised manuscript (line 574-575).

*3. Minor: Paragraph 252-259 is a bit hard to follow.*

Response: We have added more evidences and made corrections to this paragraph to make it easier to understand. We compared the protein structure of Omega-1 and four *S. japonicum* T2 ribonucleases and found that they all contained the ribonuclease_T2 domain (S3 Fig). Additionally, we cited a new reference (reference 31) that reported all copies of *S. mansoni* omega-1 were specifically expressed in eggs which was consistent with our result. Please check the revised manuscript (lines 325-341) for more details.

*4. Minor: I’m not sure I agree with the statement that “AS has been investigated in S. japonicum schistosomula and adult worms [69,70], but these analyses were based on the highly fragmented V1 version genome (SjV1) that will cause the loss of information.” The PRJEA34885 genome is quite fragmented, but prjna520774 is comparatively not too bad compared to other Schistosoma genome assemblies. And the “fragmentation” in genomes more often occur in highly repetitive gene-poor regions, so* *a fair few alternative splicing events can be correctly inferred even in a highly fragmented genome.*

Response: Thanks for your comment. It’s worth noting that AS investigations in schistosomula and adult worms were based on the *Sj*V1 genome. The *Sj*V2 genome (prjna520774) wasn’t out at that time. We have rewritten this part into ‘AS has been investigated in *S. japonicum* schistosomula and adult worms [20,21], but they were based on the early RNA-Seq technologies, the sequencing depth and accuracy were far inferior to the one used in our study.’ (revised manuscript, line 642-645).

*5. Major: Alternative splicing patters in prjna520774 are available for anyone to browse at https://parasite.wormbase.org/jbrowse/index.html?data=%2Fjbrowsedata%2Fschistosoma_japonicum_prjna520774%2Fdata&loc=scaffold_84%3A3696001..4170000&tracks=DNA%2Ccmscan_rfam_12.2.nucleotide_match%2Crepeatmasker.repeat_region%2CGene_Models%2Cdust.low_complexity_region%2Ctrf.tandem_repeat&highlight= so what the authors have done in terms of predicting alternative splicing is not particularly new. They also have not shared the genome or gene prediction they have done, and not validated its accuracy to current versions of genes and gene models. The instinct that the authors have in going to look for alternative splicing patterns between the different life stages is great, and potentially could come up with some interesting patterns. Figure 4d is really quite neat though, I like it more and more the more I look at it.*

Response: Thanks for your comment. The AS events in *Sj*V2 were predicted by gene models and the RNA-Seq data of sporocysts, cercariae, and adult worms. We don’t know what AS events were occurred in specific life stage. In our study, we performed AS analysis in the four larval stages-eggs, miracidia, sporocysts, and cercariae. We showed the total number and different types of AS in each stage. Furthermore, we also analyzed the differential AS events between the adjacent life stages. All the tables of AS analyses were provided in the Dataset S2.

*6. Minor: 500-502 reference missing for the statement “previously detected in schistosomula”.*

Response: References have been added to the revised manuscript (line 643).

*7. Minor: 503-506 The statement “SE is thought to be the most prevalent AS type in animals, whereas IR represents the most common AS form in plants and unicellular eukaryotes [72]. Hence, our results suggest that the gene regulation pattern of S. japonicum is much closer to its mammalian hosts.” I don’t think anyone who has ever seen the S. japonicum would disagree with the authors, but this is also clearly not a unique and new finding, or even unexpected, since S. japonicum are animals, and not unicellular eukaryotes.*

Response: Thanks for your reminding. We have added more organisms for comparison. This sentence has been corrected into ‘Among all events, IR is the predominant mode of AS in plants [22], whereas ES is the major type in humans [23]. However, in the cestodes *Echinococcus granulosus* and *Echinococcus multilocularis* or the free-living flatworm *Schmidtea mediterranea*, the major type AS was IR [24,25]. Hence, our results suggest that the gene regulation pattern of *S. japonicum* is much closer to its human hosts.’ (revised manuscript, lines 680-685).

*8. Major: They just have not put their results properly into the context of the other studies of differential expression of Schistosoma egg, miracidium, sporocyst and cercaria. It would not be that hard to download and re-analyze previously published data, and more carefully go through similarities and differences. Most of their main results are either things that are already known, or relates to an unpublished genome.*

Response: We have compared our data with Gobert 2009 and Cai 2017 by manual checking. We observed that only a few of the stage-specific genes or stage-enriched genes from our data were found in Gobert 2009. E.g., aquaporin 9 and calmodulin in egg, dynein light chain 2 and plexin in sporocysts, and glutamine synthetase in cercariae. Moreover, only half of the top 25 genes enriched in the eggs and cercariae stage identified in Cai 2017 were found in our SEG or SSG lists. The comparison results were added in the revised manuscript (lines 297-298 and 328-329). Considering stem cells may contribute to the asexual reproduction in larval stages, we analyzed stem cell markers from recently published transcriptome and scRNAseq works of schistosomes [26–28] and added a new part-‘Genes associated with germinal cell and asexual reproduction in the larval stages’ in the Results and Discussion section (revised manuscript, lines 511-567). We speculate that germinal cells are existing in all larval stages and stem cells conversion may be occurred during the larval developmental transition.

*9. Minor: The data accessions cited are not yet publicly available: PRJNA719283, SRR14133806-SRR14133817. (assuming they will be)*

Response: Once this paper is allowed to be published, we will release the data immediately.

Summary and General Comments

Reviewer #1:

*1. The authors describe and compare the transcriptomes of the S. japonicum larval stages using RNAseq. This will be a welcome resource for the community. The authors go on to identify and characterise duplication events in the peptidase C1 family (members of which have important role infection) as well as examining their transcript abundances in each larval stage. Finally, the group begin to evaluate the alternative splicing across the transcriptomes.*

Response: Thank you!

*2. Whilst the analysis is good greater detail is needed in both description of the methods and in the discussion of results. The importance of some results are not well stated. Additionally, the introduction is a little brief and could describe in more detail some of the key morphological and molecular differences between larval stages to aid the non-expert reader in interpretation of their work. Whilst the work is understandable, I think it would benefit from additional copy editing for grammar/sentence structure – though this is absolutely not a major concern.*

Response: Thanks for your suggestion. We have added more detail in the description of the key morphological and molecular differences between the larval stages (revised manuscript, lines 83-102). We have tried our best to optimize the grammar/sentence structure, and we hope the revised version can make you understand better.

Reviewer #2:

*1. This is a very thoroughly analysed and nicely presented piece of work, which gives new insight into the intramolluscan transcriptome landscape of Schistosoma japonicum. I recommend publication subject to corrections of minor issues.*

Response: Thank you!

*2. My only criticism is whether the coverage of analysis is a little selective - this is always the case with papers like this, authors naturally focus on what is interesting to them, and you cannot please everybody. But I was just left wondering about expression of for example neoblast (stem cell) related genes, which are en vogue in the helminth literature recently, could the authors consider examining these to see if they inform when and where stem cell replication is occurring in the life cycle?*

Response: Thanks for your wonderful suggestion. We have compared our data to existing transcriptome and scRNAseq data set of *S. mansoni*. Considering the scRNAseq data were mainly focused on stem cells [26–28], we picked 18 stem cell markers and identified their homologs in *S. japonicum*, and then described their expression patterns in our RNA-seq data. Please find the details in ‘Genes associated with germinal cell and asexual reproduction in the larval stages’ in the Results and Discussion section (revised manuscript, lines 511-567).

*3. Line 108: Authors should note here that a transcriptome is already available for intramolluscan S. mansoni (doi: 10.1371/journal.pntd.0007013), but since that one was generated from whole snails, it highlights the novelty of the current approach in which separate ex vivo life stages have been analysed.*

Response: We have cited this important reference in the revised manuscript (line 114). This sentence has been corrected into ‘Recently, the intramolluscan transcriptomes of *S. mansoni* were investigated [29], but the samples used for RNA-Seq were infected whole snails. Thus, the landscape of gene expression in ex vivo larval stages, especially the molecular basis behind the stage transitions, is still lacking.’ (revised manuscript, lines 113-116).

*4. Line 146: Indicate volume/mass of parasite tissue used for RNA extraction from each life stage. You specify three biological replicates per library in results, I suggest mentioning that here also.*

Response: Thanks for your comment. The mass of parasite used for RNA extraction was added in the ‘RNA isolation, library preparation and sequencing’ section (revised manuscript, lines 187-189). The biological replicates information of each stage was added in the ‘Parasite material’ (revised manuscript, lines 159-160, 171-172, 177-178, and 183-184).

*5. Line 176: correct "manosoni"*

Response: Corrected.

Reviewer #3:

*1.* *In this paper, authors have done RNA-Seq of egg, miracidium, sporocyst and cercaria of S. japonicum – a zoonotic parasitic worm. It is an achievement, because these are very small, it is difficult to extract and enrich them fast enough, and in enough quantity to yield good sequencing. The authors have done 3 biological replicates of each life-stage and RNA-Seq, which has been thoroughly analysed using standard and appropriate methods. The paper aims to give new insights into an hitherto underexplored part of the S. japonicum life cycle, but I’m not sure it quite does. They should have mentioned that the same and more life stage expression profiling has been done previously by Gobert 2009* *PMC2670322 (not referenced) and Cai 2017* *PMC5223471 (referenced). Very similar studies have also been conducted for closely related Schistosoma species, and in my opinion this paper would be enriched for more carefully comparing and contrasting their results with those of previous studies. They do find their results much overlap with what already is known, which is showing that the study was probably technically quite well executed. But going into a bit more detail comparing/contrasting with other would have given them more to talk about. Or alternatively – pick the most interesting story and going in for more proper experimental validation of the different ideas raised?*

Response: We appreciate that you get to this point. We do notice the published papers (Gobert 2009 PMC2670322 and Cai 2017 PMC5223471). The sequencing methods they used are either EST or Microarray, which has lower resolution than RNA-seq. Besides, our newly assembled chromosol level of *S. japonicum* genome is expected to improve the transcriptomic analysis. In the revised manuscript, We have refenced Gobert 2009 (line 105). Nevertheless, we compared our data with Gobert 2009 and Cai 2017 by manual checking. We observed that only a few of the stage-specific genes or stage-enriched genes from our data were found in Gobert 2009. E.g., aquaporin 9 and calmodulin in egg, dynein light chain 2 and plexin in sporocysts, and glutamine synthetase in cercariae. Moreover, only half of the top 25 genes enriched in the eggs and cercariae stage identified in Cai 2017 were found in our SEG or SSG lists. The comparison results were added in the revised manuscript (lines 297-298 and 328-329). Considering stem cells may contribute to the asexual reproduction in larval stages, we analyzed stem cell markers from recently published transcriptome and scRNAseq works of schistosomes [26–28] and added a new part-‘Genes associated with germinal cell and asexual reproduction in the larval stages’ in the Results and Discussion section (revised manuscript, lines 511-567). We speculate that germinal cells are existing in all larval stages and stem cells conversion may be occurred during the larval developmental transition.

**References**

1.	Iii JJC, King RS, Cogswell A, Williams DL, Newmark PA. An Atlas for *Schistosoma mansoni* Organs and Life-Cycle Stages Using Cell Type-Specific Markers and Confocal Microscopy. PLoS Negl Trop Dis. 2011;5: e1009. doi:10.1371/journal.pntd.0001009

2.	Wangwiwatsin A, Protasio AV, Wilson S, Owusu C, Holroyd NE, Sanders MJ, et al. Transcriptome of the parasitic flatworm *Schistosoma mansoni* during intra-mammalian development. PLoS Negl Trop Dis. 2020;14: e0007743. doi:10.1371/journal.pntd.0007743

3.	Maciel LF, Morales-Vicente DA, Silveira GO, Ribeiro RO, Olberg GGO, Pires DS, et al. Weighted Gene Co-Expression Analyses Point to Long Non-Coding RNA Hub Genes at Different *Schistosoma mansoni* Life-Cycle Stages. Front Genet. 2019;10. doi:10.3389/fgene.2019.00823

4.	Wang J, Yu Y, Shen H, Qing T, Zheng Y, Li Q, et al. Dynamic transcriptomes identify biogenic amines and insect-like hormonal regulation for mediating reproduction in *Schistosoma japonicum*. Nat Commun. 2017;8: 14693. doi:10.1038/ncomms14693

5.	Grote A, Caffrey CR, Rebello KM, Smith D, Dalton JP, Lustigman S. Cysteine proteases during larval migration and development of helminths in their final host. PLoS Negl Trop Dis. 2018;12: e0005919. doi:10.1371/journal.pntd.0005919

6.	Sojka D, Hartmann D, Bartošová-Sojková P, Dvořák J. Parasite Cathepsin D-Like Peptidases and Their Relevance as Therapeutic Targets. Trends Parasitol. 2016;32: 708–723. doi:10.1016/j.pt.2016.05.015

7.	Yang Y, Wen Y jun, Cai YN, Vallée I, Boireau P, Liu MY, et al. Serine Proteases of Parasitic Helminths. Korean J Parasitol. 2015;53: 1–11. doi:10.3347/kjp.2015.53.1.1

8.	Hagerty JR, Jolly ER. Heads or tails? Differential translational regulation in cercarial heads and tails of schistosome worms. PLOS ONE. 2019;14: e0224358. doi:10.1371/journal.pone.0224358

9.	Leontovyč R, Young ND, Korhonen PK, Hall RS, Tan P, Mikeš L, et al. Comparative Transcriptomic Exploration Reveals Unique Molecular Adaptations of Neuropathogenic *Trichobilharzia* to Invade and Parasitize Its Avian Definitive Host. PLoS Negl Trop Dis. 2016;10: e0004406. doi:10.1371/journal.pntd.0004406

10.	Ram D, Grossman Z, Markovics A, Avivi A, Ziv E, Lantner F, et al. Rapid changes in the expression of a gene encoding a calcium-binding protein in *Schistosoma mansoni*. Mol Biochem Parasitol. 1989;34: 167–175. doi:10.1016/0166-6851(89)90008-X

11.	Hambrook JR, Kaboré AL, Pila EA, Hanington PC. A metalloprotease produced by larval *Schistosoma mansoni* facilitates infection establishment and maintenance in the snail host by interfering with immune cell function. PLoS Pathog. 2018;14: e1007393. doi:10.1371/journal.ppat.1007393

12.	Duvaux-Miret O, Stefano GB, Smith EM, Dissous C, Capron A. Immunosuppression in the definitive and intermediate hosts of the human parasite *Schistosoma mansoni* by release of immunoactive neuropeptides. Proc Natl Acad Sci U S A. 1992;89: 778–781. doi:10.1073/pnas.89.2.778

13.	Inbar E, Hughitt VK, Dillon LAL, Ghosh K, El-Sayed NM, Sacks DL. The Transcriptome of *Leishmania major* Developmental Stages in Their Natural Sand Fly Vector. mBio. 2017;8: mBio.00029-17, e00029-17. doi:10.1128/mBio.00029-17

14.	Anderson L, Amaral MS, Beckedorff F, Silva LF, Dazzani B, Oliveira KC, et al. *Schistosoma mansoni* Egg, Adult Male and Female Comparative Gene Expression Analysis and Identification of Novel Genes by RNA-Seq. PLoS Negl Trop Dis. 2015;9: e0004334. doi:10.1371/journal.pntd.0004334

15.	Zhang B, Wang Y, Liu J-Y. Genome-wide identification and characterization of phospholipase C gene family in cotton (*Gossypium* spp.). Sci China Life Sci. 2018;61: 88–99. doi:10.1007/s11427-017-9053-y

16.	Xie T, Chen C, Li C, Liu J, Liu C, He Y. Genome-wide investigation of WRKY gene family in pineapple: evolution and expression profiles during development and stress. BMC Genomics. 2018;19: 490. doi:10.1186/s12864-018-4880-x

17.	Li Y, Song Q, Zhang Y, Li Z, Guo J, Chen X, et al. Genome-wide identification, characterization, and expression patterns analysis of the SBP-box gene family in wheat (*Triticum aestivum* L.). Sci Rep. 2020;10: 17250. doi:10.1038/s41598-020-74417-x

18.	Zhu B, Luo F, Shen Y, Yang W, Sun C, Wang J, et al. *Schistosoma japonicum* cathepsin B2 (*Sj*CB2) facilitates parasite invasion through the skin. PLoS Negl Trop Dis. 2020;14: e0008810. doi:10.1371/journal.pntd.0008810

19.	Liu M, Ju C, Du X-F, Shen H-M, Wang J-P, Li J, et al. Proteomic Analysis on Cercariae and Schistosomula in Reference to Potential Proteases Involved in Host Invasion of *Schistosoma japonicum* Larvae. J Proteome Res. 2015;14: 4623–4634. doi:10.1021/acs.jproteome.5b00465

20.	Piao X, Hou N, Cai P, Liu S, Wu C, Chen Q. Genome-wide transcriptome analysis shows extensive alternative RNA splicing in the zoonotic parasite *Schistosoma japonicum*. BMC Genomics. 2014;15: 715. doi:10.1186/1471-2164-15-715

21.	Wang X, Xu X, Lu X, Zhang Y, Pan W. Transcriptome Bioinformatical Analysis of Vertebrate Stages of *Schistosoma japonicum* Reveals Alternative Splicing Events. PloS One. 2015;10: e0138470. doi:10.1371/journal.pone.0138470

22.	Chaudhary S, Khokhar W, Jabre I, Reddy ASN, Byrne LJ, Wilson CM, et al. Alternative Splicing and Protein Diversity: Plants Versus Animals. Front Plant Sci. 2019;10. doi:10.3389/fpls.2019.00708

23.	Wang ET, Sandberg R, Luo S, Khrebtukova I, Zhang L, Mayr C, et al. Alternative isoform regulation in human tissue transcriptomes. Nature. 2008;456: 470–476. doi:10.1038/nature07509

24.	Resch AM, Palakodeti D, Lu Y-C, Horowitz M, Graveley BR. Transcriptome Analysis Reveals Strain-Specific and Conserved Stemness Genes in *Schmidtea mediterranea*. PLoS ONE. 2012;7: e34447. doi:10.1371/journal.pone.0034447

25.	Liu S, Zhou X, Hao L, Piao X, Hou N, Chen Q. Genome-Wide Transcriptome Analysis Reveals Extensive Alternative Splicing Events in the Protoscoleces of *Echinococcus granulosus* and *Echinococcus multilocularis*. Front Microbiol. 2017;8: 929. doi:10.3389/fmicb.2017.00929

26.	Wang B, Collins JJ III, Newmark PA. Functional genomic characterization of neoblast-like stem cells in larval *Schistosoma mansoni*. eLife. 2013;2: e00768. doi:10.7554/eLife.00768

27.	Wang B, Lee J, Li P, Saberi A, Yang H, Liu C, et al. Stem cell heterogeneity drives the parasitic life cycle of *Schistosoma mansoni*. eLife. 2018;7: e35449. doi:10.7554/eLife.35449

28.	Diaz Soria CL, Lee J, Chong T, Coghlan A, Tracey A, Young MD, et al. Single-cell atlas of the first intra-mammalian developmental stage of the human parasite *Schistosoma mansoni*. Nat Commun. 2020;11: 6411. doi:10.1038/s41467-020-20092-5

29.	Buddenborg SK, Kamel B, Hanelt B, Bu L, Zhang S-M, Mkoji GM, et al. The in vivo transcriptome of *Schistosoma mansoni* in the prominent vector species *Biomphalaria pfeifferi* with supporting observations from *Biomphalaria glabrata*. PLoS Negl Trop Dis. 2019;13: e0007013. doi:10.1371/journal.pntd.0007013
