# Supplementary material for: Comparative transcriptome profiles of Schistosoma japonicum larval stages: Implications for parasite biology and host invasion
Source: PLoS Negl Trop Dis. 2022 Jan 13;16(1):e0009889. doi: 10.1371/journal.pntd.0009889 (PMC8791509; doi:10.1371/journal.pntd.0009889)
Supplement: Attachment — Submitted filename: Response to Reviewers.docx [file pntd.0009889.s019.docx]

Reviewer Comments to Author:
*1. The most important problem is that the reference genome is still not available, which makes this work impossible to replicate in its current state. The authors now provide an accession number for a BioProject, but that is currently not visible - I guess under embargo pending a genome paper becoming available. but in the context of the current manuscript, we have no guarantee of the availability of these data on any defined timeframe. I think the authors need to either make the bioproject publicly available before this paper can be accepted, or make these data available for review somewhere (e.g. FTP or, preferably, an alternative data repository).*

Response: According to your request, we have uploaded the genome data to the OneDrive cloud (link: <https://1drv.ms/u/s!Anh7o-mU0R69bubqj99sxS4KhWE?e=QqWNmh>, password: pntdSjv3) for review. The link is valid for 20 days. Notably, our genome paper is still in the critical review stage, so we want the data to be kept confidential.

*2. There are two new sections of text (added in response to reviewer comments) that attempt to explain how the highest-level RNA-seq differences observed correlate with the expected biological differences between different developmental stages of schistosomes, but they are so high-level as to not really do anything useful, and rather repetitive between the two. There is already a much better description of the life-cycle in the Introduction. I would just delete these two bits, and leave in a sentence like: (e.g. at line 394): "Since the parasite at each stage shows distinct biological characteristics, we attempted to* *identify genes expressed specifically at each stage, or significantly more highly expressed at each stage. We thus defined.". At line 147 I would delete the first sentence 'Miracidium embryo was developing inside the immature egg', as it doesn't really tell us anything about the pattern of gene expression expected.*

Response: Thanks for your suggestions. All corrected.

*line 40-42: The expression profile of the stem cell markers revealed the potential germinal cell conversion during the larval development --> The expression profile of stem cell markers revealed potential germinal cell conversion during larval development*

*lines 53-54: of the S. japonicum larval stages and identifies a set of genes that might involve in the intermediate and definitive host invasion. --> of S. japonicum larval stages and identifies a set of genes that might be involved in intermediate and definitive host invasion.*

*lines 77-78: Additionally, it indicated that different populations of germinal cells maybe existed in the larval stages. -> Additionally, it indicated that different populations of germinal cells may exist in the larval stages.*

*line 159 - maybe -> 'may be'*

*line 186 - to be absolutely clear, I think the sentence about rabbit infections could do with an 'each' at the end.*

*line 196, were served as a biological replicate -> were used as a biological replicate*

*lines 208, 220: were served as one replicate. -> were used as one replicate*

*line 212- after three times of washing -> after washing three times*

*line 246: After the clusters generation -> After cluster generation*

*line 261: The Principal Component Analysis -> Principal Components Analysis*

*line 415: T2 ribonuclease was one of the top 25 genes enriched in eggs showed in the previous study - would be better as--> T2 ribonuclease was identified as one of the top 25 most highly enriched genes in eggs in a previous study.*

*line 652: The dot line --> The dotted line*

*line 712 : study the schistosome stem cells, -> study schistosome stem cells,*

*line 725: Then we described the expression files of -> Then we described the expression profiles of*

*line 738: relative high expression -> relatively high expression*

*line 929: schistosomula -> schistosomulae*

*line 932, 954: IR --> RI*

*line 986: the penetration of the miracidia to the snail. -> the penetration of the snail by the miracidia.*

Response: All corrected.

*line 952-954: Could differences in AS event type frequency be due to technical differences between the different experiments, rather than species differences?*

Response：Thanks for your comment. Our RNA-seq was based on the Next-generation sequencing (NGS) technology, which was the same as the one used in human, plants or *Echinococcus* [1–3]. With NGS technology, RNA-seq has quickly become widely used to identify AS events with high resolution. There may be some differences in the prediction accuracies of AS based on different sequencing platforms, but less in AS event type frequency. So the differences in AS event type frequency was mainly due to the species differences.

*line 992 - 994: Expression profile of the stem cell markers revealed that different populations of germinal cells were existed in the larval stages. -> The expression profile of stem cell markers revealed that different populations of germinal cells exist in larval stages.*

Response：Corrected.

References:

1.	Chaudhary S, Khokhar W, Jabre I, Reddy ASN, Byrne LJ, Wilson CM, et al. Alternative Splicing and Protein Diversity: Plants Versus Animals. Front Plant Sci. 2019;10. doi:10.3389/fpls.2019.00708

2.	Wang ET, Sandberg R, Luo S, Khrebtukova I, Zhang L, Mayr C, et al. Alternative isoform regulation in human tissue transcriptomes. Nature. 2008;456: 470–476. doi:10.1038/nature07509

3.	Liu S, Zhou X, Hao L, Piao X, Hou N, Chen Q. Genome-Wide Transcriptome Analysis Reveals Extensive Alternative Splicing Events in the Protoscoleces of *Echinococcus granulosus* and *Echinococcus multilocularis*. Front Microbiol. 2017;8: 929. doi:10.3389/fmicb.2017.00929
